# The release of lindane from contaminated building materials

**DOI:** 10.1007/s11356-014-2742-x

**Published:** 2014-03-21

**Authors:** Konstantin Volchek, Geneviève Thouin, Wenxing Kuang, Ken Li, F. Handan Tezel, Carl E. Brown

**Affiliations:** 1 Environment Canada, 335 River Road, Ottawa, ON K1A0H3 Canada; 2 Sustainable Development Technology Canada, 45 O’Connor Street, Suite 1850, Ottawa, ON K1P 1A4 Canada; 3 Department of Chemical and Biological Engineering, University of Ottawa, 161 Louis Pasteur, Ottawa, ON K1N 6N5 Canada

**Keywords:** Lindane, γ-hexachlorocyclohexane, Building materials, Inhalation, Dechlorination, Isomerisation, TWA, Decontamination

## Abstract

The release of the organochlorine pesticide lindane (γ-hexachlorocyclohexane) from several types of contaminated building materials was studied to assess inhalation hazard and decontamination requirements in response to accidental and/or intentional spills. The materials included glass, polypropylene carpet, latex-painted drywall, ceramic tiles, vinyl floor tiles, and gypsum ceiling tiles. For each surface concentration, an equilibrium concentration was determined in the vapour phase of the surrounding air. Vapor concentrations depended upon initial surface concentration, temperature, and type of building material. A time-weighted average (TWA) concentration in the air was used to quantify the health risk associated with the inhalation of lindane vapors. Transformation products of lindane, namely α-hexachlorocyclohexane and pentachlorocyclohexene, were detected in the vapour phase at both temperatures and for all of the test materials. Their formation was greater on glass and ceramic tiles, compared to other building materials. An empiric Sips isotherm model was employed to approximate experimental results and to estimate the release of lindane and its transformation products. This helped determine the extent of decontamination required to reduce the surface concentrations of lindane to the levels corresponding to vapor concentrations below TWA.

## Introduction

Lindane is an organochlorine pesticide, which was used extremely broadly for decades to control insects in crops, disinfect soil, treat lice in human and animal, and in a number of other applications (CEC 2006). The use of lindane has been, however, drastically reduced over the last 15 years. This pesticide was found toxic, persistent, and bioaccumulative, and it is now controlled by the Stockholm Convention on Persistent Organic Pollutants (Vijgen et al. 2011). Most industrialized nations have banned the manufacturing and/or application of lindane; however, it is still used in some countries, such as the US and Canada, for pharmaceutical purposes and some others, such as Mexico, allow a limited agricultural use (CEC 2006). Even in countries that have banned the large-scale use of lindane, episodes of air pollution by lindane have been reported (Yao et al. 2010).

Lindane is actually a common name of the γ-isomer of 1,2,3,4,5,6-hexachlorocyclohexane (HCH). Technical formulations of lindane contain other isomers of HCH, namely α, β, δ, and ε; however, γ-HCH is a much stronger insecticide than any of the other isomers, and it is the active ingredient of the formulations (Manonmani 2011). Isomers of HCH are known to transform into each other in certain environmental conditions (Malaiyandi and Shah 1984; Walker et al. 1999). HCH may also undergo dechlorination with pentachlorocyclohexene (PCCH) being one of key degradation products (Bhatt et al. 2009; Li et al. 2011). As shown further in this paper, α-HCH and PCCH were discovered in the tests as transformation products of γ-HCH. Table 1 summarizes the main physicochemical characteristics of γ-HCH, α-HCH, and PCCH. It also lists a time-weighted average (TWA) for each of the isomers. NIOSH (2010) defines TWA as an average concentration of a contaminant in the air to which workers may be exposed without adverse effect over a period for up to 10-h workday during a 40-h workweek.

**Table 1:**
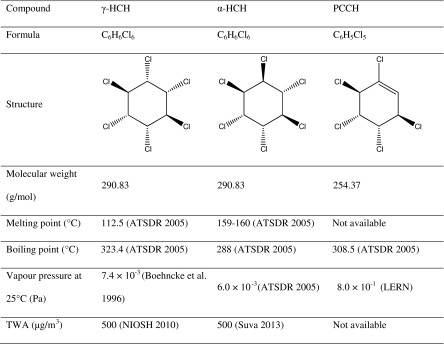
Properties of polychlorocyclohexanes Data from ATSDR 2005, ChemSpider 2013, NIOSH 2010, and Suva 2013

It is estimated that approximately 600,000 tons of lindane were manufactured and consumed across the world between 1950 and 2000 (Vijgen et al. 2011). While a vast majority of it was utilized, there are still many pesticide storage facilities remaining across the world that contain lindane. Some of these facilities were reported to become sources of environmental pollution by lindane (Dvorská et al. 2012). Furthermore, Manonmani (2011) estimated that approximately 4.8 million tons of lindane manufacturing waste may be present that still contains some γ-HCH. All of this presents a serious environmental problem that must be addressed sooner rather than later. In particular, old lindane storage facilities may require decontamination so that they no longer pose a threat.

Decontamination is a process whose purpose is to bring the level of contamination to or below what is considered a safe level. In some cases, decontamination may be accomplished by simply removing hazardous substances or involve a more elaborate physicochemical (or biological) treatment. As far as pesticide storage facilities are concerned, their decontamination may be necessary even if they will no longer be used. For example, cleanup of walls, floors, and other elements will be required to ensure a safe work environment for demolition crews.

Lindane health and environmental hazard has been viewed mainly through its ingestion pathway, since lindane was used on crops, a key element of a food chain (CEC 2006; Vijgen et al. 2011). While this prioritization is understandable, inhalation is the main risk factor to workers in case of building decontamination or demolition (Shaurette 2010). It is worth mentioning that while building materials are the objects of decontamination, it is the air quality that defines inhalation hazard. From this prospective, it is important to draw correlations between the concentrations of lindane (and/or other contaminants which may be present) on the surface and the concentrations in the vapor phase.

Lindane interactions with soil (Chen and Zhu 2005a, b; Chen and Zhu 2005a; Goss et al. 2008; Krishna and Philip 2008; Manonmani 2011; Phillips et al. 2004) and other solid matrices (Delle Site 2001; Xiao et al. 2004) have been studied quite extensively. Shitta-Bey (2009) investigated the partitioning of lindane between air and dust in indoor environments. At the same time, very little information could be found about the interactions of lindane with building materials that would help better understand the associated hazards. The objective of this work was, thus, to study the behavior of lindane on materials surface and its effect on lindane concentration in the air. This was viewed as a tool in assessing and predicting the inhalation hazard related to the contamination of building materials by lindane. TWA was used as a reference occupational standard. NIOSH (2010) lists TWA for γ-HCH at 500 μg/m^3^.

The experimental approach used in this study was to spike coupons of the test materials with known surface concentrations of lindane, place them in a closed container, let the system reach equilibrium, and then analyze the corresponding lindane concentrations in the air. Correlations would be drawn better in the vapor and the surface concentrations so that the latter could be linked to TWA.

## Materials and methods

### Materials

The γ-HCH of analytical standard grade (purity >99.9 %) was purchased from Sigma-Aldrich Chemical Co. in a powder form and was used in the experiments without additional purification. Test coupons were cut from six different commonly used construction materials: RCR woven polypropylene carpet, Grenada gypsum/cellulose fiber ceiling tiles, Terra Grigio unglazed ceramic (porcelain) floor tiles, Leah Harbor polyvinyl chloride floor tiles, CIL latex-painted CGC gypsum drywall (wallboard), and borosilicate glass. Each coupon had a square shape with 5 cm × 5 cm linear dimensions. Carpet, ceiling tiles, ceramic floor tiles, and vinyl tiles were purchased from RONA, Inc. Drywall and white flat latex paint were purchased from Home Depot Canada. Glass samples were obtained from Fisher Scientific Canada.

Polyvinyl fluoride 10 L Tedlar® bags (E.I. Dupont de Nemours & Company) were used as test containers. Tedlar® was chosen as being suitable for storing vapour samples of organochlorine pesticides (Budnik et al. 2010). Tenax® TA tubes (6 mm × 11.5 cm) were used to collect lindane vapour samples for their analyses. The tubes were purchased from Supelco, Inc.

### Experimental procedures

Each test coupon measured 5 cm × 5 cm was spiked with a known mass of γ-lindane powder, ranging from 1 mg to 1 g, i.e., to produce surface concentrations raging from 0.04 to 40 mg/cm^2^. This was done by spreading the powder over the entire coupon’s surface using a spatula. Each sample was placed into a Tedlar air-sampling bag. Bags were then sealed with a TISA-455 electric impulse sealer (TEW Electric Heating Equipment Company) and filled with 10 L of nitrogen gas (N_2_). Nitrogen rather than air was used to eliminate the possibility of lindane reacting with oxygen of the air; however, parallel experiments with air generated results similar to those involving nitrogen. Studies of lindane adsorption on various solid matrices (Chen and Zhu 2005a, b; Krishna and Philip 2008) revealed that adsorption equilibrium could be achieved within 10 to 30 h, depending on test conditions. In this study, bags were left at 20 or 40 °C for 4 days (96 h) which was deemed to be sufficient to reach equilibrium. Vapour samples were then pumped through Tenax tubes from the bags with an HFS 513A Hi Flow Sampler (Gilian) at a flow rate of 100 mL/min. Sampling duration was 10 min at 20 °C and 2 min at 40 °C. Three different samples were taken from the same bag for each set of conditions to assess the repeatability. These repeats are included as error bars in the figures shown in Results and discussion.

### Analyses

All analyses were carried out on a DynaTherm MTDU 900/ACEM 900 thermodesorption system coupled with an Agilent 6890 gas chromatograph (GC)/5973N mass spectrometry detector (MSD) system. An aliquot of 1 μL of 75 ppm of hexachlorobenzene was added to each Tenax tube before analysis as an internal standard. The GC was equipped with a 30-m HP-5MS column with an inside diameter of 0.25 mm and 0.25-μm film. The column was directly connected to the thermodesorption system.

The GC temperature program was as follows: 90 °C, held for 0.2 min, ramped at 15 °C/min to 150 °C, and finally ramped at 35 °C/min to 280 °C. The MSD was operated in scan mode (40 to 400 amu) with a solvent delay of 1.3 min. Quantitation ion was 181 amu for γ-HCH, α-HCH, and PCCH, with retention times of 5.1, 4.8, and 3.3 min, respectively. The detection limit was 0.25 ng. The relative standard deviation of six injections for internal standard was less than 5 %.

## Results and discussion

### Vapour-phase concentration profiles and transformation product distribution

Predetermined mass amounts of γ-HCH were placed on glass and carpet coupons to produce surface concentrations. Vapour-phase concentrations, expressed in μg of substance per m^3^ of vapor in the headspace were determined 96 h later at 20 and 40 °C. In addition to γ-HCH, two of its transformation products were also detected in the vapour phase: α-HCH and PCCH. Both products were not detected in samples of the initial γ-HCH, and it was known that Tedlar bag material would not react with γ-HCH. It was thus concluded that these products were generated in reactions of γ-HCH on surface of the test materials. This correlates with earlier reports on α-γ isomerization of HCH (Malaiyandi and Shah 1984; Walker et al. 1999) and its dechlorination into PCCH (Bhatt et al. 2009; Li et al. 2011).

**Fig. 1:**
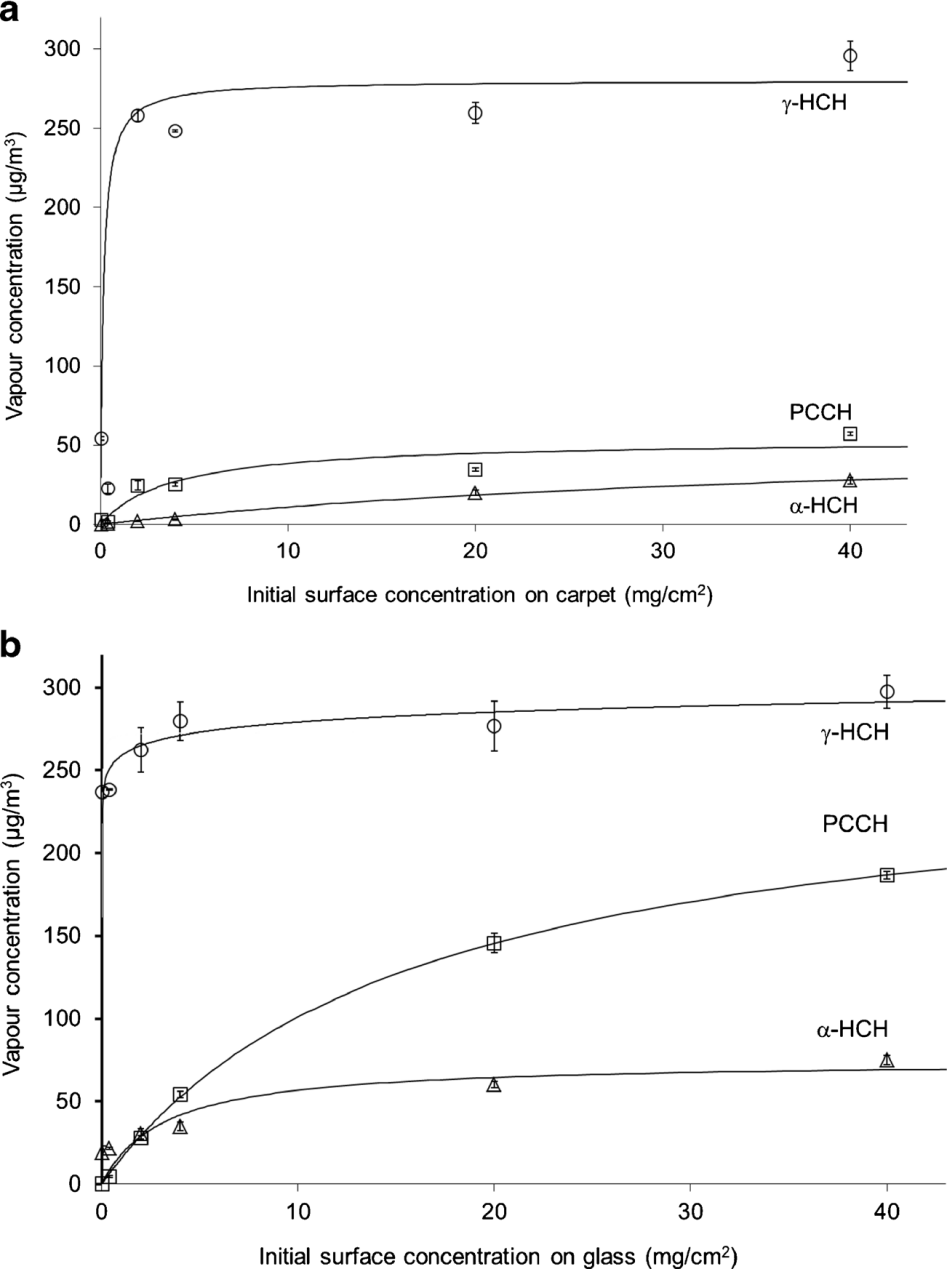
Concentrations of γ-HCH, α-HCH, and PCCH in the vapour phase as functions of the initial γ-HCH concentration on surface after 96 h of release from carpet (**a**) and glass (**b**) at 20 °C. *Points* represent experimental data and *curves* represent hyperbolic fits according to Eq. 3

**Fig. 2:**
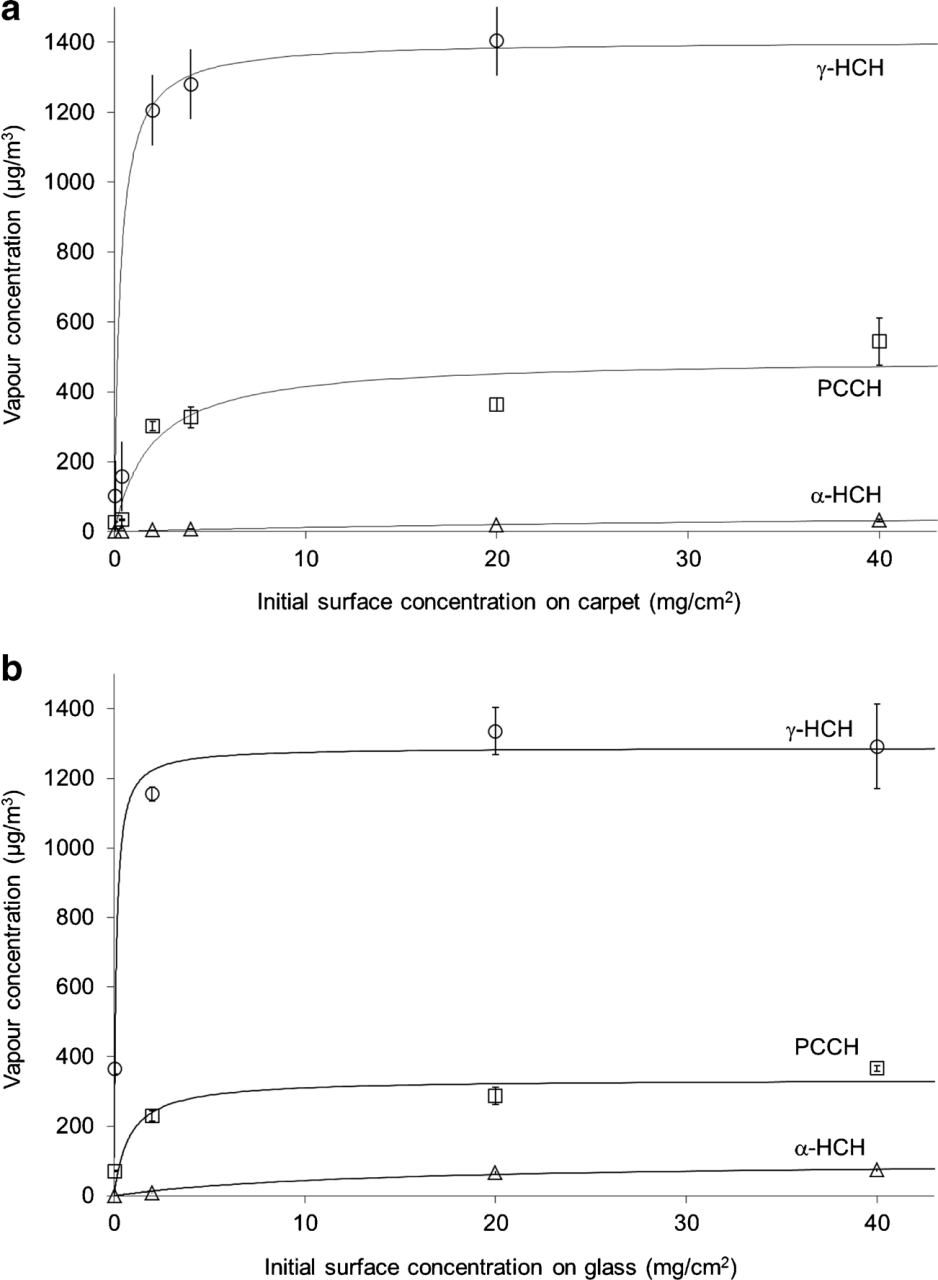
Concentrations of γ-HCH, α-HCH, and PCCH in the vapour phase as functions of the initial γ-HCH concentration on surface after 96 h of release from carpet (**a**) and glass (**b**) at 40 °C. *Points* represent experimental data and *curves* represent hyperbolic fits according to Eq. 3

The release of γ-HCH, α-HCH, and PCCH at 20 °C is shown in Fig. 1a for carpet and Fig. 1b for glass. Accordingly, Figs. 1a and 2b depict respective test data for carpet and glass at 40 °C. For both temperatures, an increase in initial surface concentration of γ-HCH first resulted in a steep increase in vapour concentration. Further increase in the surface concentration had a lesser effect on the vapour concentration which eventually formed plateaus, as the system was approaching the vapour saturation state. The observed plateau concentrations were compared to the saturation concentrations of γ-HCH calculated based on Eq. 1 given by Boehncke et al. (1996). Here, the saturation vapour pressure of γ-HCH *P*_*sat*_ (Pa) depends on the temperature *T* (K) between 292 and 326 K as follows:

1 $$ {P}_{sat}= \exp \left(34.53-\frac{11,754}{T}\right) $$

This results in *P*_*sat*_ equal to 3.75 × 10^−3^ Pa at 20 °C and to 4.87 × 10^−2^ Pa at 40 °C. *P*_*sat*_ was then used to calculate the saturation concentrations of γ-lindane in the vapour phase at 20 and 40 °C, using the ideal gas law as follows:

2 $$ {C}_{sat}=\frac{M\cdot {P}_{sat}}{R\cdot T} $$

where *C*_*sat*_ (g/m^3^) is the saturation vapour concentration, *M* (g/mol) is the molecular weight of γ-lindane, *P*_*sat*_ (Pa) is the saturation vapour pressure of γ-lindane, *R* (8.314 J/K/mol) is the universal gas constant, and *T* (K) is the temperature. This gives a saturation vapour-phase concentration of 450 μg/m^3^ at 20 °C and 5,500 μg/m^3^ at 40 °C. The plateau concentrations were thus below the saturation concentration values calculated from respective vapour pressures at 20 °C (270 μg/m^3^ observed vs. 450 μg/m^3^calculated) and at 40 °C (1,250 μg/m^3^ observed vs. 5,500 μg/m^3^calculated). The plateau vapour-phase concentration of γ-HCH at 20 °C is approximately a half of TWA value of 500 μg/m^3^, while at 40 °C is two and a half times higher.

A simple hyperbolic fit (Eq. 3) was applied to approximate the experimental data as follows:

3 $$ {C}_g=\frac{a_1\cdot C}{a_2+C} $$

where *C*_*g*_ (μg/m^3^) is the concentration of the compound in the vapour phase, *a*_1_ (μg/m^3^) is the maximum concentration of the compound in the vapour phase, *a*_2_ (mg/cm^2^) is an empirical constant, and *C* (mg/cm^2^) is the initial concentration of γ-lindane on the surface.

Results of the hyperbolic fits are presented in Table 2. The steep slopes of curves for γ-HCH on glass (Figs. 1b and 2b) suggest that the release of γ-HCH from glass is likely driven by the sublimation, which is the mechanism of transfer of γ-HCH from the original solid form into the vapour form (Giustini et al. 1998; Vecchio 2010). In comparison, the effects of the interactions with the building material, such as adsorption/desorption, play a lesser role. The curves for carpet shown in Figs. 1a and 2a have more gradual slopes than those for glass, indicating that more γ-HCH is retained by carpet and less is released in the vapour phase. This can be explained by the fact that the organic carbon-reach polypropylene fibers have a stronger affinity to lindane molecules, compared to the affinity of inorganic glass. This finding correlates with a relative sorption of lindane on different materials reported by Chen and Zhu (2005a, b); lindane is better adsorbed on matrices that have carbon-related functional groups.

**Table 2:** Hyperbolic fit parameters for experimental data in Figs. 1 and 2

Compound	Surface	Temperature	Hyperbolic Fit	
			*a*_1_ (μg/m^3^)	*a*_2_ (mg/cm^2^)
γ-HCH	Glass	20 °C	270	0.007
		40 °C	1,300	0.11
	Carpet	20 °C	300	0.97
		40 °C	1,800	1.5
α-HCH	Glass	20 °C	260	16
		40 °C	100	13
	Carpet	20 °C	58	43
		40 °C	70	49
PCCH	Glass	20 °C	75	3.2
		40 °C	330	0.79
	Carpet	20 °C	53	3.8
		40 °C	510	2.0

The hyperbolic fit given by Eq. 3 was also applied to the concentrations of α-HCH and PCCH as a function of the initial surface concentration of γ-HCH. Generally, the curves for α-HCH and PCCH had more gradual slopes than respective curves for γ-HCH. This was likely due to the fact that γ-HCH vapors had to be produced first followed by the formation of by-products in interactions with surface materials. Tichenor et al. (1991) reported that building materials act as sinks for the vapors of organic materials. This would be applicable to both γ-HCH and to its transformation products. The products would be formed and then partially retained by the building material rather than being fully released into air.

As seen in Fig. 1a, b, equilibrium vapour concentration of γ-HCH was similar for both glass and carpet at 20 °C. On the other hand, the equilibrium vapour concentrations of the transformation products were much higher for glass than for carpet. Therefore, the overall toxicity in the vapour phase at 20 °C was higher for glass than for carpet. There are two implications from this finding. First, even if the initial concentration of γ-HCH on the surface is the same, the resulting toxicity of surrounding air may differ for different materials. Second, measuring the concentration of γ-HCH alone to assess the toxicity may not be sufficient as the transformation by-products add to it. Transformation products should be taken into account too. In all tests, except those on glass at 20 °C and the surface concentration of γ-HCH less than 3.4 mg/cm^2^, PCCH was present in the vapour phase in considerably higher concentrations compared to α-HCH and was therefore the primary transformation product of γ-HCH. For example, the ratio of vapour concentrations of PCCH to α-HCH on carpet at 20 °C (Fig. 1a) and in a range of surface concentrations from 10 to 40 mg/cm^2^ was approximately between 2 and 8. In comparison, this ratio was approximately between 25 and 60 at 40 °C.

At 20 °C and the initial surface concentration of 40 mg/cm^2^, the concentration of γ-HCH in the vapour phase reached approximately 60 % of its TWA value of 500 μg/m^3^, for both carpet (Fig. 1a) and glass (Fig. 1b). As indicated earlier, Swiss occupational standards (Suva 2013) list the same value of 500 μg/m^3^ for α-HCH as the North American (USA and Canada) TWA for γ-HCH (NIOSH 2010). No occupational standard could be found for PCCH. It was reported that the toxicity of PCCH is significantly less than that of γ-lindane (Bhatt and Kumar 2009); however, when γ-HCH degrades to PCCH, it also produces the toxic hydrogen chloride gas HCl (1 mol of HCl per mole of γ-HCH). Just for estimation purposes in this study, the TWA for γ-HCH was applied total polychlorocyclohydrocarbons, including γ-HCH, α-HCH, and PCCH. At 20 °C, the total concentration of polychlorocyclohydrocarbons in the vapour phase reached approximately 75 and 110 % of the TWA from carpet coupons and glass coupons, respectively. At 40 °C, the concentration of γ-HCH was approximately three times the TWA, in test with both glass and carpet. The concentration of total polychlorocyclohydrocarbons was thus up to five times the TWA and reached approximately 5 % of the Immediately Dangerous to Life or Health (IDLH) level of 50,000 μg/m^3^. NIOSH (2010) defines IDLH as “any condition that poses an immediate or delayed threat to life or that would cause irreversible adverse health effects or that would interfere with an individual’s ability to escape unaided from a permit space.”

For each tests, the masses of γ-HCH and its transformation products in the vapour phase were estimated as their concentrations in the vapour phase at equilibrium, as collected in the Tenax tubes, multiplied by the volume of the Tedlar air-sampling bag. The total mass of all the polychlorocyclohydrocarbons in the vapor phase was much smaller than the mass of γ-HCH initially deposited on the surface. Experimental results showed that it never exceeded 0.45 % of the initial mass of γ-HCH. In other words, only a small fraction of the total γ-HCH participated in mass transfer between air and surface. This should, however, be viewed in the context of vapour adsorption by the coupon material. As discussed earlier, the adsorption of organic vapours was likely to be higher on the carpet than on the glass. As shown in Figs. 1 and 2, at lower surface concentrations of γ-HCH of 5 mg/cm^2^ or less, the difference in vapour concentrations over glass and over carpet was quite visible due to the contribution of adsorption. At higher concentrations, the difference was minimal, if any existed at all. Mass transfer at high initial surface concentration was likely to be dominated by sublimation rather than adsorption/desorption, so the effect of surface materials was diminished.

### Release isotherms

In order to estimate vapour concentrations at different surface concentrations and temperatures, Sips isotherm model was used as described by Do (1998). The correlation between the equilibrium concentrations on surface and in vapor phase is given using the following equation:

4 $$ {C}_s=\frac{C_{sm,0}\cdot \exp \left[\chi \cdot \left(1-\frac{T}{T_0}\right)\right]\cdot {\left\{{b}_0\cdot \exp \left[\frac{Q}{R\cdot {T}_0}\cdot \left(\frac{T}{T_0}-1\right)\right]\cdot {C}_v\right\}}^{\left[{n}_0+\alpha \left(1-\frac{T_0}{T}\right)\right]}}{1+{\left\{{b}_0\cdot \exp \left[\frac{Q}{R\cdot {T}_0}\cdot \left(\frac{T}{T_0}-1\right)\right]\cdot {C}_v\right\}}^{\left[{n}_0+\alpha \left(1-\frac{T_0}{T}\right)\right]}} $$

where *C*_*s*_ is the equilibrium surface concentration (mg/cm^2^), *C*_*sm*,0_ is the saturation surface concentration (mg/cm^2^) at the reference temperature *T*_0,_ *χ* is a dimensionless constant parameter, *T* is the temperature (K), *T*_0_ is the reference temperature set at 293 K, *b*_0_ is the adsorption constant (m^3^/μg) at the reference temperature T_0_, *Q* is the heat of adsorption (J/mol), *R* is the universal gas constant equal to 8.314 J/(K mol), *C*_*v*_ is the equilibrium vapour-phase concentration (μg/m^3^), *n*_0_ is a dimensionless parameter characterizing the system heterogeneity at the reference temperature T_0_, and *α* is a dimensionless constant parameter.

**Fig. 3:**
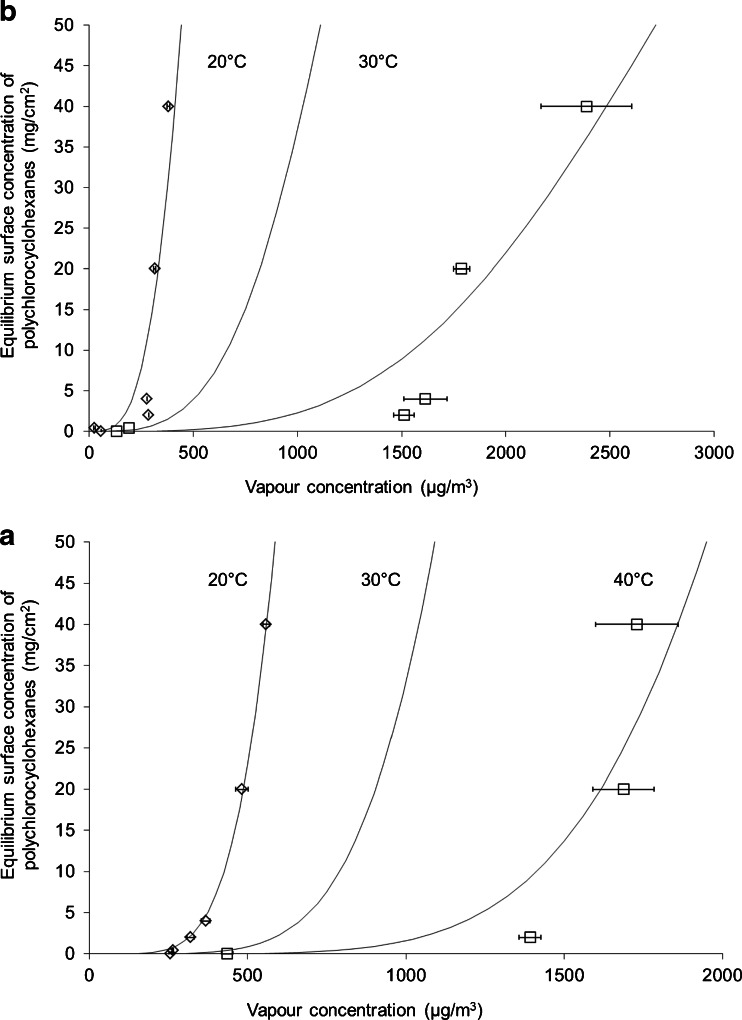
Equilibrium isotherm for the release of polychlorocyclohexanes from carpet (**a**) and glass (**b**) using the Sips isotherm model. *Points* represent the experimental data at 20 and 40 °C. *Curves* represent model predictions at 20, 30, and 40 °C

Figure 3 depicts the experimental results and the predicted values of the equilibrium vapour concentrations of total polychlorocyclohydrocarbons as function of the surface concentrations of γ-HCH on carpet (a) and glass (b), respectively. Figure 3 presents the data in the form of adsorption isotherms where the *x*-axis corresponds to the vapour concentration and the *y*-axis corresponds to the surface concentration. Since only a very small fraction of γ-HCH participated in mass transfer between the surface and the vapour phase, the initial surface concentration of γ-HCH was used in Fig. 3 as the equilibrium surface concentration of polychlorocyclohydrocarbons.

The Sips isotherm parameters for glass and carpet at 20 and 40 °C are given in Table 3. The calculated heat of adsorption *Q* was greater for carpet (56,000 J/mol) than for glass (45,000 J/mol). This confirmed that γ-lindane and likely polychlorocyclohydrocarbons in general adsorbed stronger on the organic-based carpet than on inorganic glass. The calculated surface saturation concentration *C*_*sm*,0_ was also slightly higher on carpet (390 mg/cm^2^) than on glass (360 mg/cm^2^). Overall, Fig. 3 demonstrates a good correlation between the experimental data and those calculated using the Sips model.

**Table 3:** Sips isotherm parameters for total polychlorocyclohexanes

Surface	*C*_*sm*,0_ (mg/cm^2^)	*b*_0_ (m^3^/μg)	*n*_0_	α	*Q* (J/mol)	χ
Glass	360	0.0012	5.4	0.0	45,000	1.2
Carpet	390	0.0013	3.4	0.00020	56,000	14

### Application of Sips isotherms to assess decontamination requirements

**Fig. 4:**
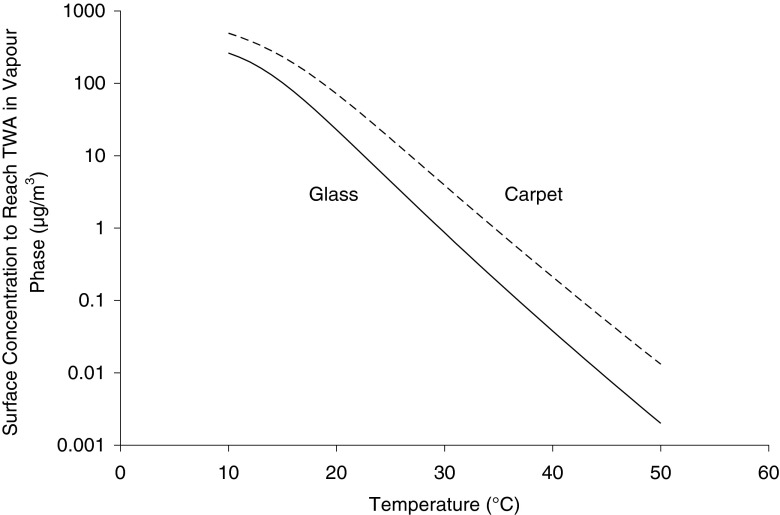
Equilibrium surface concentration (*C*_*s*_) corresponding to the TWA of 500 μg/m^3^ in the vapour phase calculated from Eq. 4 for temperatures ranging from 10 to 50 °C

The Sips isotherm model was used to determine the surface concentration (*C*_*s*_) corresponding to a vapour-phase concentration (*C*_*v*_) equal to the TWA. As indicated in Vapour-phase concentration profiles and transformation product distribution, the TWA value of 500 μg/m^3^ for γ-lindane was employed as the TWA for total polychlorocyclohydrocarbons as *C*_*v*_. The corresponding *C*_*s*_ values were then calculated at temperatures ranging from 10 to 50 °C using Eq. 4 and parameters from Table 3. The calculated *C*_*s*_ values for glass and carpet are shown in Fig. 4 as functions of temperature. These *C*_*s*_ concentrations corresponded to the maximum surface concentrations of respective compounds at which safe working environment was still possible. In other words, if the surface concentrations exceeded *C*_*s*_, then the concentration in the vapour phase would exceed the TWA. Surface decontamination would therefore be necessary.

In addition to helping to decide whether the decontamination is required or not, *C*_*s*_ also helps to estimate the extent of decontamination required to attain the TWA in the air. For example, if the surface concentrations exceed *C*_*s*_ by a factor of ten, then the required decontamination should reach at least 90 % removal of the contaminant from the surface.

Several hypothetical scenarios for glass and carpet are provided in Table 4. Based on the Sips model Eq. 4 and the parameters in Table 3 determined for glass and carpet, minimum decontamination efficiencies required to revert to a safe environment were estimated based on the TWA. The minimum decontamination efficiency (MDE) was defined as follows:

5 $$ \mathrm{MDE}=100\cdot \left(1-\frac{C_{TWA}}{C}\right) $$

where MDE (%) is the minimum decontamination efficiency, *C*_*TWA*_ (mg/cm^2^) is the equilibrium surface concentration (*C*_*s*_) that corresponds to the equilibrium vapour concentration (*C*_*v*_) of the TWA, calculated from Eq. 4, and *C* (mg/cm^2^) is the initial surface concentration.

**Table 4:** Minimum decontamination efficiency (*MDE*) required for glass and carpet depending on the level of contamination and the temperature

Initial surface concentration of γ-HCH, (mg/cm^2^)		Temperature at which *TWA* is attained (°C)	*MDE* (%)				
			120 °C	25 °C	30 °C	35 °C	40 °C
Glass	0.1	36	-	-	-	-	62
	1	29	-	-	14	82	96
	10	22	-	56	91	98	99.6
	100	15	77	96	99	99.8	>99.99
Carpet	0.1	42	-	-	-	-	-
	1	34	-	-	-	11	79
	10	27	-	-	61	91	98
	100	19	28	83	96	99	99.8

At the same initial surface concentration *C*, the minimum decontamination efficiency MDE depends only on *C*_*TWA*_. Having the same TWA value for total polychlorocyclohydrocarbons, higher *C*_*TWA*_ values were found for carpet than for glass from Eq. 4 at a given temperature as shown in Fig. 4. A lower MDE was therefore required for carpet compared to that required for glass.

For both carpet and glass, higher decontamination efficiencies would be needed at higher temperatures. This is due to the fact that equilibrium vapour concentrations increase as temperature rises. As a result, if surface decontamination was carried out at a lower temperature and it resulted in a vapour concentration below the TWA, further decontamination could still be required if building temperature was expected to rise. For example, for a surface concentration on carpet equal to 10 mg/cm^2^, the TWA is attained at 27 °C. This means that when temperature exceeds 27 °C, decontamination would be required. The higher the temperature, the greater the decontamination effort required. At 30 °C, at least 61 % of γ-HCH must be removed from the surface so that the vapour concentration does not exceed TWA. In comparison, the removal should be at least 98 % at 40 °C to stay below TWA. This should be taken into account in cases involving old pesticide storage facilities which are not equipped with ventilation or air conditioning and where temperatures can exceed 40 °C on hot summer days.

### Screening tests on different building materials

In addition to glass and carpet, experiments were also carried out on other construction materials including ceiling tiles, painted drywall, ceramic tiles, and vinyl floor tiles at 20 and 40 °C contaminated with 4 mg/cm^2^ of γ-HCH. The concentrations of γ-lindane, α-lindane, and PCCH were measured in the vapor phase. Similarly to glass and carpet, it was assumed that the 4-day period was sufficient to reach equilibrium on those materials.

**Fig. 5:**
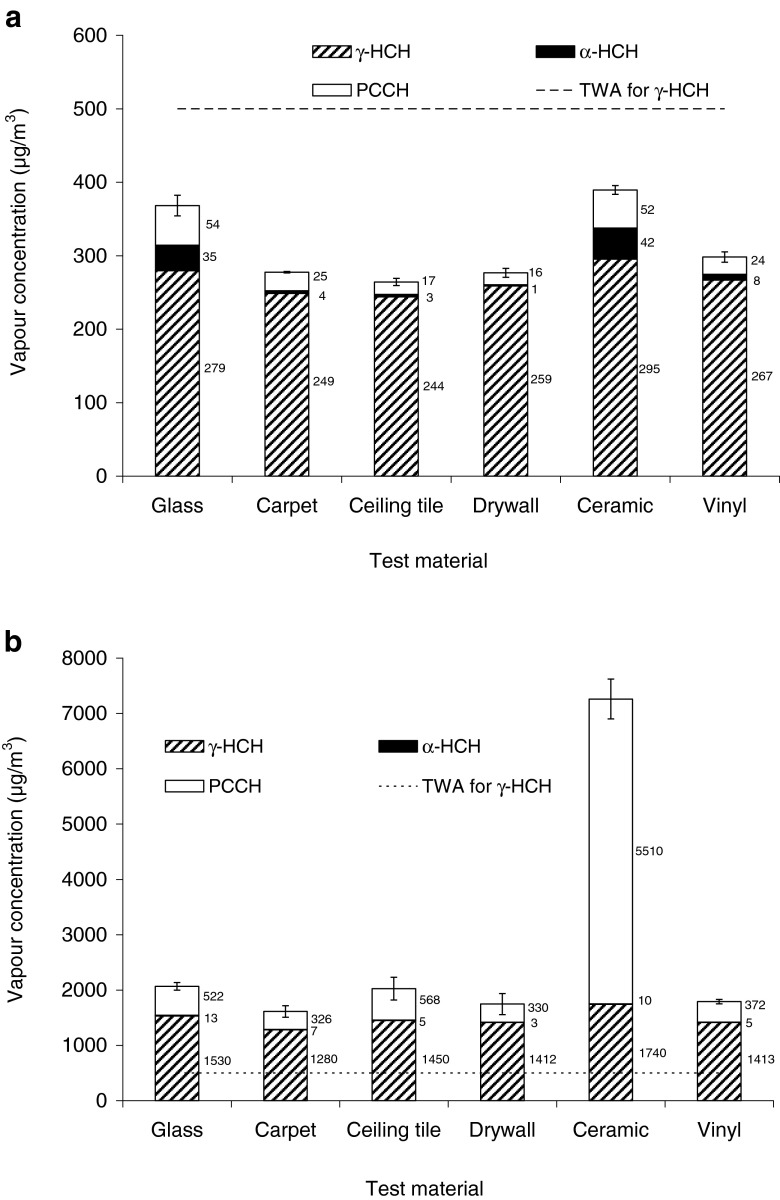
Concentrations of polychlorocyclohexanes in the vapour phase for various building materials contaminated with 4 mg/cm^2^ of γ-lindane at 20 °C (**a**) and 40 °C (**b**) after 96 h of release. *Error bars* indicate error in total polychlorocyclohexane concentrations

As shown in Fig. 5, the concentrations of γ-HCH in the vapour phase were very close for all materials, averaging at 270 μg/m^3^ at 20 °C and 1,500 μg/m^3^ at 40 °C. In comparison, the concentrations of transformation products differed more significantly. For example, the vapour concentration of PCCH for ceramic tiles at 40 °C was greater than 5,000 μg/m^3^, which was more than three times higher than the vapour concentration of γ-HCH. At the same time, the vapor concentrations of PCCH for carpet and vinyl tiles were only about 500 μg/m^3^. The high vapour-phase concentrations of PCCH detected for ceramic tiles can likely be explained by the porous surface of the tiles and the presence of metals as active sites. The latter can act as mediators of γ-HCH dihaloelimination and the formation of PCCH (Li et al. 2011). Another observation was that while both α-HCH and PCCH were detected in comparable concentrations at 20 °C, the concentrations of PCCH were far greater than those of α-HCH at 40 °C. The action of metals was more profound at a higher temperature which resulted in a major increase in PCCH concentration in the vapour phase.

## Conclusions

When lindane (γ-hexachlorocyclohexane) is spilled on building materials, lindane vapours are released in the surrounding air. The release was found to be greater at a higher temperature, mainly due to a stronger sublimation. This study showed that the concentration of lindane in the air was not just a result of its surface concentration and temperature but also depended on the type of building material. Organically rich materials such as polypropylene carpet fibers showed a stronger retention of lindane which resulted in its lower release, whereas inorganic materials such as glass demonstrated a lower retention and a higher release, respectively.

Two transformation products of lindane, α-hexachlorocyclohexane and pentachlorocyclohexene, were detected in the vapour phase. While their presence was observed in all experiments, the relative concentrations of by-products were considerably higher in tests involving inorganic building materials such as glass and especially ceramic tiles. The presence of metal oxides acting as catalysis of transformation was the likely reason of this phenomenon. In tests on ceramic tiles, the concentration of pentachlorocyclohexene in the vapour was three times as high as the concentration of lindane.

A comparison of the vapour concentrations of lindane and its products to the time-weighted average (TWA) for lindane revealed that at 20 °C, the total concentrations were below or slightly above TWA in the entire range of lindane surface concentrations. At 40 °C, the vapour concentrations exceeded TWA except at very low surface concentrations. This suggested that surface decontamination was necessary to bring vapour concentrations below TWA level.

Based on the correlations between the surface and the vapour concentrations established in this study, the minimum decontamination efficiency (MDE) was estimated for several scenarios involving the contamination of building materials by lindane. The efficiency depended on the surface concentration, type of material, and temperature. It was concluded that while a moderate decontamination effort could be sufficient to for a given building material at a lower temperature, the decontamination would have to be repeated more rigorously if temperature inside the building was set to rise.

## Nomenclature

*a*_1_: Maximum concentration in the vapour phase (μg/m^3^)
*a*_2_: Empirical constant (mg/cm^2^)
*b*_0_: Adsorption constant at reference temperature *T*_0_ (m^3^/μg)
*C*: Initial surface concentration (mg/cm^2^)
*C*_*g*_: Concentration in vapour phase (μg/m^3^)
*C*_*s*_: Equilibrium surface concentration (mg/cm^2^)
*C*_*sat*_: Saturation vapour-phase concentration (mg/m^3^)
*C*_*sm*,0_: Saturation surface concentration at reference temperature *T*_0_ (mg/cm^2^)
*C*_*TWA*_: Surface concentration corresponding to the TWA in the vapor phase (mg/cm^2^)
*C*_*v*_: Equilibrium vapour concentration (μg/m^3^)
*M*: Molecular weight (g/mol)
*MDE*: Minimum decontamination efficiency (%)
*n*_0_: Parameter characterizing the system heterogeneity at reference temperature *T*_0_ (dimensionless)
*P*_*sat*_: Saturation vapour pressure (Pa)
*Q*: Heat of adsorption heat (J/mol)
*R*: Universal gas constant (8.314 J/K mol)
*T*: Temperature (K)
*T*_0_: Reference temperature (293 K)
*TWA*: Time-weighted average (μg/m^3^)
*a*: Empirical constant (dimensionless)
*χ*: Empirical constant (dimensionless)
